# QSAR study and the hydrolysis activity prediction of three alkaline lipases from different lipase-producing microorganisms

**DOI:** 10.1186/1476-511X-11-124

**Published:** 2012-09-28

**Authors:** Haikuan Wang, Xiaojie Wang, Xiaolu Li, Yehong Zhang, Yujie Dai, Changlu Guo, Heng Zheng

**Affiliations:** 1 Key Laboratory of Industrial Fermentation Microbiology, Ministry of Education, College of Biotechnology, Tianjin University of Science and Technology, Tianjin, 300457, P. R. China; 2 College of Oceanography, Harbin Institute of Technology, Weihai, 264209, P. R. China; 3 School of Life Science and Technology, China Pharmaceutical University, Nanjing, 210009, P. R. China

**Keywords:** QSAR, GFA, Ester, Lipase activity, Vegetable oils

## Abstract

The hydrolysis activities of three alkaline lipases, L-A1, L-A2 and L-A3 secreted by different lipase-producing microorganisms isolated from the Bay of Bohai, P. R. China were characterized with 16 kinds of esters. It was found that all the lipases have the ability to catalyze the hydrolysis of the glycerides, methyl esters, ethyl esters, especially for triglycerides, which shows that they have broad substrate spectra, and this property is very important for them to be used in detergent industry. Three QSAR models were built for L-A1, L-A2 and L-A3 respectively with GFA using Discovery studio 2.1. The models equations 1, 2 and 3 can explain 95.80%, 97.45% and 97.09% of the variances (*R*^*2*^_*adj*_) respectively while they could predict 95.44%, 89.61% and 93.41% of the variances (*R*^*2*^_*cv*_) respectively. With these models the hydrolysis activities of these lipases to mixed esters were predicted and the result showed that the predicted values are in good agreement with the measured values, which indicates that this method can be used as a simple tool to predict the lipase activities for single or mixed esters.

## Introduction

Lipases are defined as triacylglycerol acylhydrolases (E.C. 3.1.1.3) that catalyze the hydrolysis of oils and fats at the oil–water interface to free fatty acids and glycerol. Microbial lipases have been proven to be useful biocatalysts for obtaining chiral, non-racemic compounds. Lipase from *Burkholderia cepacia* can efficiently the reaction of catalyze hydrolysis, alcoholysis, transesterification, aminolysis, acidolysis, and esterification [1-3]. In order to improve the usefulness of lipases as biocatalysts, an understanding of the lipase application in daily life is needed. They directly or indirectly form an integral part of the industries ranging from food, pharmaceuticals [4], and detergents [5,6] to organic synthesis, cosmetics [7], leather, and tea industries [8]. However, the single biggest market for their use is in detergents where their functional importance lies in the removal of fatty residues in laundry, dishwashers, and for cleaning clogged drains [9]. Though the lipase function is usually connected with enzyme activity, the higher enzyme activity, the better washing performance is, sometimes the washing performance is not fully consistent with the lipase activity. One reason is that there are different methods for the determination of lipase activity. At present, the lipase activity is usually determined by titrimetric methods [10], spectrophotometry [11], nephelometry and turbidimetry [12], electric conductivity [13], and so on. And each of them based on a specific property of the lipase reaction system, which leads to the different activity measuring values for the same lipase. The other reason is that the substrates in detergency ability evaluation are different from that in the determination of lipase activities [14]. In the washing performance evaluation, the substrates used are usually mixture of different fats or oil, for example, lipase decontamination capability was measured using emulsified olive oil as the substrate [15,16]. Decontamination capability is related to lipase activity, while animal fat and plant oils are main oil pollution daily in our lives. The main components of these oil pollutions are triglyceride, diacylglycerol, free fatty acid, etc. However, the substrate used in the determination of lipase activity is usually a pure matter, the difference in substrates result in the difference between the washing performance and the activity of lipase. There are different lipase activities for different substrates, which results from the differences in substrate composition and structure. A lipase with better detergency ability should have higher hydrolysis ability to a broad spectrum of esters. In order to obtain comprehensive understanding of the lipase activity and substrate spectrum, the substrates with various composition and structure are required to evaluate them, further, a quantitative structure and activity relationship should be built. There are some studies on this aspect, for example, there are two distinct modeling strategies for predicting lipase activity highlights: structure-based approach and data-driven approach. The structure-based models start with a known active site structure of the lipase [17-19] and then identify the preferred substrates based on conformation, charge, and other force field calculations [20,21]. On the other hand, data driven models such as quantitative structure–activity relationship (QSAR) approach develops a mathematical relationship between the enzyme activity and structural descriptors of substrates using available experimental data. In context of lipases, such QSAR approach has been reported in predicting the substrate specificity [22] and enantioselectivity of a lipase in esterification/trans-esterification reactions [23]. However, there are few reports on the systematic evaluation of the lipase detergency ability using different substrates existed in oil spill.

Previously, three kinds of lipase from the soil collected from the Bay of Bohai, P. R. China was found by our laboratory including *Burkholderia cepacia* L-A1 [24], *Acinetobacter johnsonii* L-A2 [25,26] and *Acinetobacter calcoaceticus* L-A3 [27]. They have highly stability in the presence of various oxidizing agents, some commercial detergents and alkaline protease. The three enzymes hydrolyzed a wide range of oils and showed a high level of lipase activity in hydrolyzing glyceride. In order to systematic evaluate its ability to hydrolyze different esters including some usually existed in edible oils and fats, this study derived some quantitative structure and activity relationships (QSARs) between the experimental results and structural parameters important for the substrate specificity of *Burkholderia cepacia* L-A1, *Acinetobacter johnsonii* L-A2 and *Acinetobacter calcoaceticus* L-A3 towards triglyceride, ethyl oleate, methyl laurate and allyl phenylacetate, etc. Meanwhile, this study will be useful for developing a standard for lipase evaluation with their detergency ability.

## Materials and methods

### Lipase-producing strains

Alkaline lipase-producing microorganisms were isolated from the Bay of Bohai, P.R. China and they were numbered as *Burkholderia cepacia* L-A1, *Acinetobacter johnsonii* L-A2 and *Acinetobacter calcoaceticus* L-A3, respectively. Refined, edible vegetable oils were purchased locally. Glycerol tripalmitate from Alfa Aesar Chemical Co., LTD (Tianjin, China). Methyl hexadecanoate, methyl myristate, methyl laurate, methyl linoleate, ethyl tetradecanoate, ethyl palmitate, ethyl tetradecanoate and ethyl linoleate were from TCI (Shanghai, China). Triarachidin, ethyl palmitate, ellyl phenylacetate, eripalmitin were from Tokyo Chemical Industry Company (Japan). Methyl oleate, methyl gallate, mlyceryl monostearate, glycerol trioleate, ethyl oleate, ethyl stearate and allyl phenylacetate were bought from Sinopharm Medicine Holding Co., Ltd (Tianjin, China).

### Lipase activity determination

Lipase activity was determined based on the method described by Nahas with some modifications [28]. The substrate was dispersed in 2% (w/v) polyvinyl alcohol to form 20% (v/v) emulsion prepared by homogenizing using a top-drive homogenizer (FSH-2 adjustable high-speed homogenizer, Jiangsu Zhengji Instruments Co., Ltd., China) for 5 min and pH was adjusted to 8.0. The reaction mixture contained 4 ml of the substrate, 5 ml of PBS, and 1 ml (0.1g/ml) of crude lipase solution. After incubation at 30°C for 1 h, the reaction was stopped by the addition of 10 ml acetone/ethanol (1:1, v/v). The resulting mixture was titrated with 0.05 M NaOH until 10.5 of the end point pH was reached. Blanks were obtained with the same volume of 2% (w/v) polyvinyl alcohol and lipase samples were boiled for 10 min and the activities were expressed as μ mol free fatty acids released. Determinations were done in duplicate and the lipase activity was obtained as follows:

(1) $$X=\frac{\left(B-A\right)\times c}{0.05}\times 50\times \frac{1}{60}\times n$$

*X*, enzyme activity,U/g (U/ml).

*B*, sample consumption volume of standard sodium hydroxide solution for titration, ml.

*A*, blank sample consumption volume of standard sodium hydroxide solution for titration, ml.

*c*, standard sodium hydroxide concentration, mol/L.

0.05, conversion factor of sodium hydroxide concentration of standard solution.

50, 1ml sodium hydroxide solution (0.05 mol/L) equivalent to 50μmol fatty acid.

1/60, the reaction time of 60 min with 1 min count.

In this study, the 17 esters commercially availed listed in Additional file 1: Table S1 were used as substrates to examine 3 lipase activities, the ester hydrolytic activity data of three lipases determined using spectrophotometry were also listed.

### Generation of the 3D structure of the esters

The ester series were further subjected to molecular modeling studies using ChemBioOffice Software version 11 [29]. The 2D structure of the ester compounds was drawn in ChemBioDraw Ultra version 11 and then copied to Chem 3D Ultra version 11 to create the three-dimensional (3D) model. These structures were then subjected to energy minimization using molecular mechanics (MM2). The minimized molecules were further subjected to optimization via the Austin model 1 (AM1) method using the closed-shell (restricted) wave function of the Gamess [30].

### Descriptors for QSAR

More than 120 physiochemical properties of the esters used as descriptors for QSAR construction were obtained using the “Calculate Molecular Properties” module of the Discovery Studio 2.1 package [31]. These descriptors include 2D (*AlogP, Molecular_SurfaceArea, Num_RotatableBonds, Num_H_Donors,Molecular_Weight, Kappa_1* topological descriptors such as *CIC, CHI_3_C, IAC_Mean, BIC,IC, IAC_Total* and *SIC*, etc.) and 3D (*Jurs descriptors, Dipole, MolecularVolume* and *shadowindices*, etc.) parameters. All the definition of the descriptors can be seen in the help of DS2.1. The lipase activity in *A* U/ml was converted to the logarithmic scale before used for subsequent QSAR analyses as the response variable.

### QSAR model development

The obtained QSAR models which are developed from the training set should be validated using new esters for checking the predictive ability of the developed models. Thus the original data set is divided into training and test sets for QSAR model development and validation respectively. The ability of a model to predict accurately the target property of compounds that were not used for the model development is based on the fact that a molecule which is structurally similar to the training set molecules will be predicted well because the model has captured features that are common to the training set molecules and is able to find them in the new molecule [32]. In our study, the whole data set (n =16) was divided into training (n =12) and test (n =4) sets by function groups. This approach (clustering) ensures that the similarity principle can be employed for the lipase activity prediction of the test set [33]. The splitting has been performed such that points representing both training and test sets are distributed within the whole descriptor space of the entire dataset, and each point of the test set has a closer point of the training set. Compared with the number of molecular physiochemical properties, the training set is comparatively very small. In order to obtain the model with statistical meaning, these properties should be cut down and the most suitable descriptors will be left for the final model. The difficult thing is how to select which properties as the most suitable descriptor set to build QSAR models. In this study, the genetic function approximation (GFA) technique was employed to deal with this problem. The principles of GFA can be seen elsewhere [34,35]. It uses the multivariate adaptive regression algorithm accompanied with the genetic algorithm (GA) to evolve population of models (each model containing a subset of variables) that best fit the training set data. With this methodology, a series of potential QSAR models (the population of organisms) are generated and tested repeatedly until an approximate optimal solution is reached finally. In this study, the QSAR models having different numbers of descriptor terms were selected by GFA and all the descriptors in the QSAR trial descriptor pool were used as linear terms. Subsequently, genetic partial least squares (G/PLS) module was employed to optimize the obtained model further.

### Statistical quality assessment and model validation method

The successful QSAR model should be robust enough to make accurate and reliable predictions of the lipase activities, thus, the obtained QSAR models from the training set should be subsequently validated. There are several methods to evaluate the quality of QSAR models. In this study, Friedman lack-of-fit (*LOF*) [36] was selected as the rule for the selection of the GFA derived equations, while correlation coefficient *R*^2^ and adjusted *R*^2^ (*R*^*2*^_*adj*_), were taken as objective functions for G/PLS [37] equations’ selection. The predictivity of generated QSAR models were finally validated using leave-one-out cross-validation *R*^2^ (*R*^*2*^_*cv*_). Because the descriptor number available normally exceeds that of the samples (training set compounds), how to prevent over-fitting of GFA is critical to the successful construction of a statistically significant QSAR model. In this study, the QSAR models having different numbers of descriptor terms were selected by GFA and all the descriptors in the QSAR trial descriptor pool were used as linear terms. *LOF* is designed to control the model size and to avoid the over-fitting. The smoothing factor was set to 0.5, the optimal QSAR model was considered to be obtained when descriptors used became constant and independent of an increasing number of crossover operations. All the descriptors were used as linear terms during the GFA to generate QSAR models in the QSAR trial descriptor pool.

### QSAR model predictivity for the lipase hydrolysis ability to some natural mixed esters

In order to assess the QSAR model predictivity for the lipase hydrolysis ability to some natural mixed esters, the hydrolysis ability of the lipases L-A1, L-A2 and L-A3 to some natural oils such as soybean oil, olive oil and rapeseed oil were also determined using the technique described as 2.2. The compositions and contents of various aliphatic acids in these oils were obtained from literature with the analysis of gas chromatography/mass spectrometry (GC/MS) [38,39]. Because the composition complexity of the natural oils, the ester compositions are simply considered as the mixture of various triglycerides with three same kind of fatty acids. The esters with each content >1% are included and listed in Table 1. The hydrolysis activity of the lipase for the mixed esters is thought to be the average for each containing triglyce ation:

(2) Xmix=X¯=∑i=1nmi·yi%Mi∑i=1nyi%n·Xi

**Table 1:** Compositions of the vegetable oils

**Composition vegetable oil**	**Palmitic acid**	**Stearic acid**	**Oleic acid**	**Linolenic acid**	**Linoleic acid**
Soybean oil	11.0 ± 0.8(%)	4.5 ± 0.4(%)	20.7 ± 1.0(%)	8.9 ± 1.8(%)	54.2 ± 2.4(%)
Olive oil	14.5 ± 1.3(%)	2.5 ± 1.2(%)	70.0 ± 1.0(%)	1.5 ± 0.5(%)	12.0 ± 1.0(%)

*X*_*mix*_, the lipase activity for hydrolysis of the natural oil (U/ml).

*X*_*i*_, the lipase activity for hydrolysis of *i* oil ester component.

*y*_*i*_, the proportion of fatty acid glycerides.

*n*, the ester numbers contained in natural oil.

*m*_i_, molar fraction of each triglycerides contained in natural oil with mass fractions >1%.

QSAR model predictivity for the lipase hydrolysis ability to natural mixed esters was assessed by the comparison of the *X*_*mix*_ obtained from the experiment with that obtained from QSAR models.

## Results and discussion

### Activity comparison of three lipases

**Figure 1:**
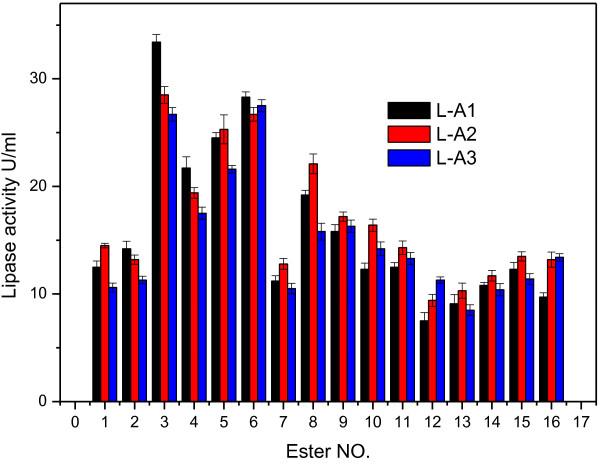
The hydrolysis activity of three lipases for different esters.

The activities of three lipases include L-A1, L-A2 and L-A3 from our laboratory toward different fatty acid methyl and ethyl esters, and fatty acid glycerides are shown in Figure 1. It can be seen that three lipases all have the ability to catalyze the hydrolysis of the test esters, especially for triglycerides, which shows that they have a broad substrate spectra, and this property is very important for them to be used in detergent industry. On the other hand, each of three lipases has its own characteristics. Compared with other two lipases, L-A1 gave good hydrolysis activities for triglycerides with the highest activity of 33 U/ml for glycerol trioleate. L-A2 shows better catalysis spectrum because it gave comparatively better hydrolysis activity for most test esters though the highest activity is not as high as L-A1 for glyceride. Generally, the substrates order according to the hydrolysis abilities of three lipases are triglyceride > monoglyceride >other esters.

### QSAR Modeling with 2D and 3D combined set of descriptors

In order to obtain the optimum descriptor set for constructing the QASR models and omit the insignificant descriptors, the GFA protocol in DS 2.1 was employed. The linear term was used for the model development with Friedman *LOF* smoothness parameter of 0.5 and the population size of 1500. The obtained QSAR models were then further treated with G/PLS and the models on the descriptors from DS 2.1 are as follows (Eq. 3, 4 and 5 are obtained the equations for L-A1, L-A2 and L-A3):

(3) $$\begin{array}{c} LogX_{\text{L}-\text{A}1}=\;1.197-0.1134\times ALogP_{M}R\\ \;+0.0307\times Molecular\_Volume-0.0330\\ \;\times Molecular\_PolarSASA-0.5881\\ \;\times Shadow\_XYfrac+0.2295\\ \;\times Jurs\_PPSA\_3 \end{array}$$

The sample number *N* = 12; *LOF* = 0.0048; *R*^2^ =0.9833; *R*^*2*^_*adj*_ =0.9694; R_cv_^2^ =0.889; *F* = 51.18

(4) $$\begin{array}{c} LogX_{L-A2}=1.966+0.0572\times Molecular\_Weight\\ \;-1.129\times CHI\_0-0.2433\times Dipole\_Y\\ \;-1.4035\times <Shadow\_XYfrac-0.524612>\\ \;+0.0167\times <56.961-Jurs\_PNSA\_1 \end{array}$$

The sample number *N* = 12; *LOF* = 0.0029; *R*^2^ =0.9861; *R*^*2*^_*adj*_ = 0.9745; *R*^*2*^_*cv*_ =0.954; *F* = 84.98

(5) $$\begin{array}{c} LogX_{\text{L}-\text{A}3}=\;3.0049-0.1796\times CHI\_1+0.2427\\ \;\times Dipole\_X-52.022\times Jurs\_FNSA\_3\\ \;-107.06\times Jurs\_FPSA\_3+0.0194\\ \;\times Shadow\_XY \end{array}$$

The sample number *N* = 12; *LOF* = 0.0024; *R*^2^ =0.9841; *R*^*2*^_*adj*_ =0.9709; *R*^*2*^_*cv*_ =0.898; *F* = 74.44

All these descriptors included in the models and their values for 12 esters accompanied with the activities from experiments and the prediction of the obtained models are listed in Tables 2, 3 and 4 respectively.

**Table 2:** Observed and predicted L-A1 activities, physiochemical properties of different substances from DS 2.1 used for the construction of QSAR models

**Substance**	***ALogPMR***	***Molecular_Volume***	***Molecular_PolarSASA***	***Shadow_XYfrac***	***Jurs_PPSA_3***	***Log*** **L-A1** ***(Obs)***	***Log*** **L-A1** ***(Pred)***	**Residual**
Training set								
1	51.297	122.79	49.521	0.623	17.109	1.097	1.084	0.013
3	296.502	793.01	115.261	0.185	58.06	1.389	1.388	0.001
4	77.398	204.42	49.521	0.677	18.406	0.875	0.902	−0.027
5	72.65	194.13	49.521	0.584	18.693	1.283	1.244	0.039
7	272.246	710.35	115.261	0.263	55.106	0.875	0.871	0.004
8	93.287	229.8	49.521	0.438	23.066	1.09	1.092	−0.002
9	103.309	283.66	120.524	0.645	32.625	1.336	1.341	−0.005
10	95.802	251.41	49.521	0.534	21.828	1.124	1.131	−0.007
12	96.919	247.3	49.521	0.44	22.368	1.033	1.057	−0.024
13	63.448	169.09	49.521	0.69	16.925	1.049	1.041	0.008
14	81.852	218.14	49.521	0.696	20.233	1.199	1.228	−0.029
15	86.6	227.4	49.521	0.677	19.962	0.959	0.923	0.036
Test set								
2	91.054	239.07	49.521	0.696	21.797	1.158	1.084	0.074
6	102.636	253.81	49.521	0.64	23.527	1.452	1.127	0.325
11	241.29	651.35	115.261	0.394	48.528	1.097	1.058	0.039
16	42.667	111.47	156.026	0.251	27.008	0.987	1.191	−0.204

^a^ Obs, observed.

^b^ Pred, predicated.

**Table 3:** Observed and predicted L-A2 activities, physiochemical properties of different Substances from DS 2.1 used for the construction of QSAR models

**Substance**	***Molecular_Weight***	***CHI_0***	***Dipole_Y***	***Shadow_XYfrac***	***Jurs_FPSA_1***	***LogL-A2*** ***(Obs)***	***LogL-A2*** ***(Pred)***	**Residual**
Training set								
1	176.212	9.519	0.059	0.623	0.677	1.161	1.158	0.003
3	885.432	45.786	0.001	0.263	0.896	0.973	0.98	−0.007
4	358.556	18.59	0.135	0.645	0.882	1.288	1.311	−0.023
5	975.639	50.029	−0.166	0.185	0.94	1.403	1.396	0.007
7	214.344	11.356	0.917	0.69	0.901	1.107	1.107	0.005
8	256.424	13.477	0.943	0.677	0.912	0.973	0.995	−0.022
9	270.451	14.184	0.199	0.696	0.918	1.236	1.205	0.031
10	294.472	15.598	0.054	0.438	0.865	1.215	1.207	0.008
12	242.397	12.77	0.223	0.584	0.91	1.344	1.367	−0.023
13	284.477	14.891	0.949	0.677	0.919	1.013	1.002	0.011
14	310.515	16.305	1.094	0.44	0.901	1.068	1.074	−0.006
15	312.53	16.305	0.934	0.534	0.926	1.236	1.215	0.021
Test set								
2	322.525	15.525	1.153	0.251	1.332	1.121	1.084	0.037
6	807.32	38.64	−0.077	0.669	0.911	1.427	1.127	0.300
11	184.146	6.813	1.384	0.394	1.392	1.155	1.058	0.097
16	298.504	14.63	0.905	0.696	3.655	1.121	1.191	−0.07

^a^ Obs, observed.

^b^ Pred, predicated.

**Table 4:** Observed and predicted L-A3 activities, physiochemical properties of different Substances from DS 2.1 used for the construction of QSAR models

**Substance**	***CHI_1***	***Dipole_X***	***Jurs_FNSA_3***	***Jurs_FPSA_3***	***Shadow_XY***	***Log*** **L-A3** ***(Obs)***	***Log*** **L-A3** ***(Pred)***	**Residual**
Training set								
1	6.326	0.67	−0.042	0.041	62.211	1.025	1.028	−0.003
3	31.028	1.397	−0.019	0.029	280.042	1.053	1.051	0.002
4	12.202	1.297	−0.037	0.04	127.108	1.243	1.239	0.004
5	34.028	0.379	−0.013	0.028	342.582	1.334	1.337	−0.003
7	7.308	−0.244	−0.024	0.031	75.615	1.021	1.037	−0.016
8	8.808	−0.173	−0.018	0.029	90.705	1.053	1.012	0.041
9	9.308	0.926	−0.02	0.03	95.404	1.212	1.218	−0.006
10	10.308	0.67	−0.023	0.033	109.456	1.152	1.146	0.006
12	8.308	0.898	−0.021	0.031	86.059	1.199	1.202	−0.003
13	9.808	−0.171	−0.016	0.028	100.225	0.929	0.948	−0.019
14	10.808	−0.019	−0.017	0.029	113.043	1.017	1.011	0.006
15	10.808	0.109	−0.015	0.028	111.62	1.004	1.015	−0.011
Test set								
2	19.308	1.67	−0.052	0.032	105.063	1.053	1.084	−0.0309
6	7.308	0.898	−0.089	0.055	261.979	1.439	1.127	0.3123
11	9.608	−0.271	−0.096	0.092	119.779	1.124	1.058	0.0658
16	11.808	−0.119	−0.019	0.011	57.29	1.127	1.191	−0.0639

^a^ Obs, observed.

^b^ Pred, predicated.

In this study, *R*^2^, *R*^*2*^_*adj*_, *R*^*2*^_*pre*_ and *R*^*2*^_*cv*_ were employed to evaluate the obtained models. Eq.3, 4 and 5 can explain 96.94%, 97.45% and 97.09% of the variances (*R*^*2*^_*adj*_) respectively while they could predict 88.9%, 95.4% and 89.8% of the variances (*R*^*2*^_*cv*_) respectively [40]. *F* >*F*_(a=0.05)_ showes that the models are those for a (non-multiplicity-corrected) confidence level of 0.95. It can be seen from Equation 3 that *Molecular_Volume and Jurs_PPSA_3* have positive contribution to the bioactivity of the lipase. However, *Molecular_PolarSASA, ALogP_MR and Shadow_XYfrac* have the negative effect on the bioactivities of the lipase L-A1.

The standardized regression coefficient for each variable is 54.54, 39.42, 6.048, 0.6085 and 19.85 respectively. Therefore, the relative importance of the descriptors according to their standardized regression coefficients is in the following order:

*ALogP_MR*>*Molecular_Volume*>*Jurs_PPSA_3*>>*Molecular_PolarSASA* >*Shadow_XYfrac*.

It was found that *ALogP_MR, Molecular_Volume* and *Jurs_PPSA_3* play the key role for the bioactivity of lipase L-A1. L-A1 tends to catalyze the hydrolysis of the esters with high *ALogP_MR* and *Jurs_PPSA_3* values. For example, glycerol trioleate has the highest *Molecular_Volume* and comparatively higher *Jurs_PPSA_3* values. And they counteract the negative contribution of *ALogP_MR* to L-A1 bioactivity, which make L-A1 possess the highest activity of 33.4U/ml.

For Eq.4, it can be found that *<56.961 −Jurs_PNSA_1> and Molecular_Weight* have positive contribution to the bioactivity of the lipase. However, *CHI_0, Dipole_Y and <Shadow_XYfrac−0.5246>* have the negative effect on the bioactivities of the lipase. The relative importance of the descriptors according to their standardized regression coefficients is in the following order:

*CHI_0 > Molecular_Weight> >* *Dipole_Y > <Shadow_XYfrac −* *0.524612> > <56.961 −* *Jurs_PNSA_1 >* (The standardized regression coefficient for each variable is 105.19, 105.75, 0.8094, 0.7227 and 0.0081 respectively). From this equation, it was found that L-A2 tends to hydrolyze glycerides with higher values of *Molecular_Weight*.

For Eq.5, the standardized regression coefficient for *CHI_1, Dipole_X*, *Jurs_FNSA_3, Jurs_FPSA_3, and Shadow_XY* is 13.68, 1.174, 3.802, 4.022 and 13.91 respectively. It can be seen that the relative importance of the descriptors is as follows:

*Shadow_XY*>*CHI_1*>*Jurs_FPSA_3*>*Jurs_FNSA_3*>*Dipole_X*.

Thus, *Shadow_XY* and *CHI_1* play the key roles in determining the lipase activity. *Jurs_FPSA_3, Jurs_FNSA_3* and *CHI_1* have the opposite contribution to the lipase activity. The dimension of the actual lipase activity value is determined by the one with higher values. For example, substrate 5 has a far higher value of *Shadow_XY* than that of *Dipole_X,* which makes L-A3 possesses comparatively higher bioactivity for it.

**Figure 2:**
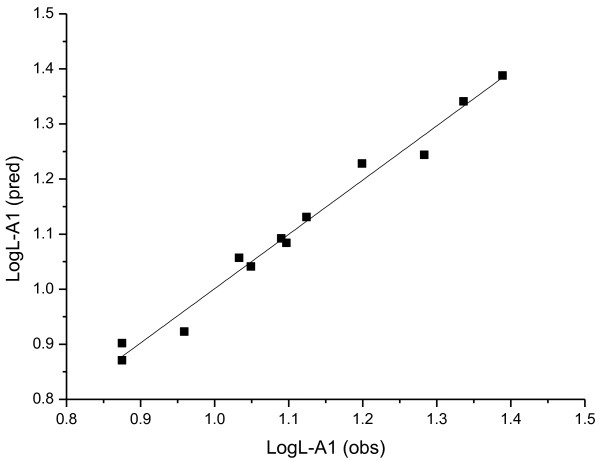
The plot of observed vs. predicted L-A1 activities of different esters in Table 2 with Equation 3.

**Figure 3:**
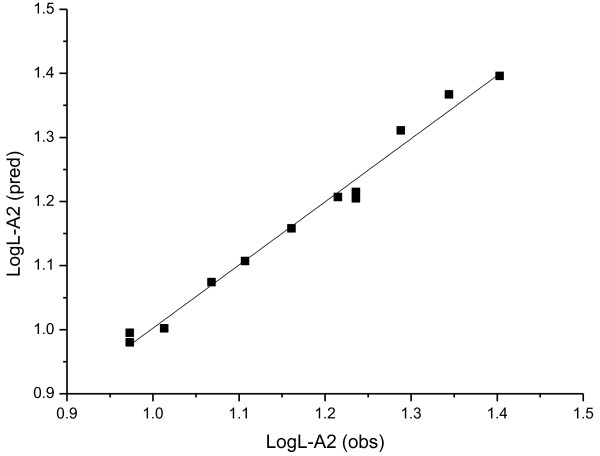
The plot of the observed *LogL-A2* vs. the predicted data with Equation 4.

**Figure 4:**
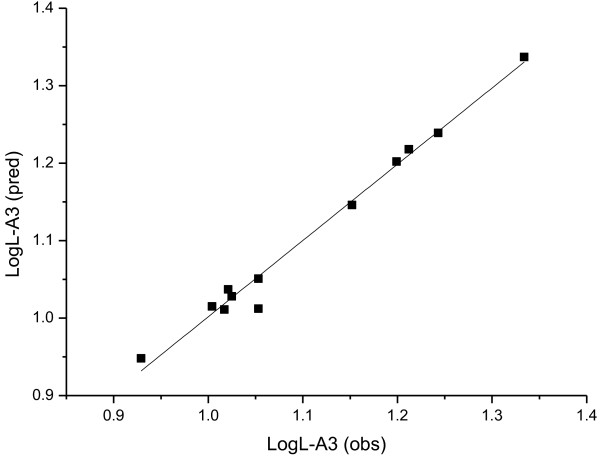
The plot of the observed *Log*L-A3 vs. the predicted data with Equation 5.

The plot of the observed lipase activities vs. the predicted data of the training set is shown in Figures 2, 3 and 4. It can be seen that the predicted data by this model is in accordance with the experimental results, which shows the good predictivity of the three models.

In order to evaluate the predictivities of these models, the four esters listed in Tables 2, 3 and 4 were used as test set and their activities were predicted with the three models were listed in Tables 2, 3 and 4.

### Prediction for the hydrolysis activity to vegetable oils

In order to predict the activities of three lipases to hydrolyze the natural oils, two vegetable oils, soybean oil and olive oil were selected as the objected oils. In order to simplify their composition, we considered that the oils are the mixture of various homotriglycerides. The esters with each content >1% and the lipase activities acquired from experiments and prediction of the QSAR models are included and listed in Table 5.

**Table 5:** Measured and predicted lipase activities for olive oil and soybean oil

**Nature oil**	**Predicted value activity(U/ml)**			**Measured value activity(U/ml)**		
	**L-A1**	**L-A2**	**L-A3**	**L-A1**	**L-A2**	**L-A3**
Olive oil	25.83	27.86	26.43	27.53	26.52	27.47
Soybean oil	28.32	26.65	29.45	29.21	28.43	28.38

It can be seen that they have good prediction for the hydrolysis ability of three lipases. For example, the predicted values of L-A1, L-A2 and L-A3 are 25.83 U/ml, 27.86 U/ml and 26.43 U/ml which is concord well with the measured values of 27.53 U/ml, 26.52 U/ml and 27.47 U/ml respectively. This result shows that these QSAR models not only can predict the lipase activity for one fat acid ester, but they can be used to predict the lipase activity for hydrolysis the natural oils composed of mixture of different esters.

## Conclusion

In this study, three QSAR models for lipases L-A1, L-A2 and L-A3 respectively were obtained using GFA algorithm in DS 2.1. The prediction of these QSAR model were evaluated by internal validation and external validation. The results showed that they have good prediction for the hydrolysis ability of three lipases it can also be used to predict and evaluate the hydrolytic activity to mixed oils.

## Competing interests

The authors declare that they have no competing interests.

## Authors’ contributions

H W, Y D and X W participated in the design, QSAR model analysis, the analysis of the ester hydrolysis results, and drafted the manuscript. X L, Y Z and C G took part in the experimental work. H Z conducted the operation of computer modeling. All authors read and approved the final manuscript.

## Supplementary Material

Additional file 1 **Table S1.** Structures of 17 esters used as substrates. Click here for file
