# Supplementary material for: QSAR study and the hydrolysis activity prediction of three alkaline lipases from different lipase-producing microorganisms
Source: Lipids Health Dis. 2012 Sep 28;11:124. doi: 10.1186/1476-511X-11-124 (PMC3567427; doi:10.1186/1476-511X-11-124)
Supplement: Additional file 1 — Table S1. Structures of 17 esters used as substrates. [file 1476-511X-11-124-S1.doc]

**Table S1** **Structures of 17 esters used as substrates**

|  | **Substrate** | **Structure** |
| --- | --- | --- |
| 1 | Allyl phenylacetate |  |
| 2 | Methyl gallate |  |
| 3 | Glycerol trioleate |  |
| 4 | Glyceryl Monostearate |  |
| 5 | Glycerol triarachidate |  |
| 6 | Glycerol tripalmitate |  |
| 7 | Methyl Laurate |  |
| 8 | Methyl myristate |  |
| 9 | Methyl hexadecanoate |  |
| 10 | Methyl linoleate |  |
| 11 | Methyl oleate |  |
| 12 | Ethyl tetradecanoate |  |
| 13 | Ethyl palmitate |  |
| 14 | Ethyl oleate |  |
| 15 | Ethyl stearate |  |
| 16 | Ethyl linoleate |  |
| 17 | 4-Nitrophenyl palmitate |  |
